# Diagnostic Value of Ileal Lesions Found during Colonoscopy with Reference to Endoscopic Indications and Findings

**DOI:** 10.3390/jcm13041161

**Published:** 2024-02-19

**Authors:** Dae Sung Kim, Ji Eun Ryu, Jieun Shin, Hoon Sup Koo, Sanghyuk Lee, Hwanhyi Cho, Jongheon Na, Kyu Chan Huh

**Affiliations:** 1 Division of Gastroenterology, Department of Internal Medicine, Konyang University College of Medicine, Daejeon 35365, Republic of Korea; 2 Healthcare Center, Konyang University Hospital, Daejeon 35365, Republic of Korea; 3 Konyang Medical Data Research Group-KYMERA, Konyang University Hospital, Daejeon 35365, Republic of Korea; 4 Department of Biomedical Informatics, College of Medicine, Konyang University, Daejeon 35365, Republic of Korea

**Keywords:** diagnostic value, terminal ileum, colonoscopy

## Abstract

The diagnostic value of ileoscopy is not well established, and its routine practice is controversial. We aimed to investigate the diagnostic value of biopsy for macroscopically abnormal lesions in the terminal ileum and to identify the association between endoscopic indications and findings and the presence of significant disease. This retrospective study included 551 patients who underwent biopsy of abnormal lesions in the terminal ileum (TI) during colonoscopy between February 2000 and June 2019. Biopsy results were analyzed in relation to the endoscopic indications and gross findings. Significant disease was defined as a case in which a specific disease was suspected or confirmed by the biopsy results, requiring additional examination or treatment. Among the 551 biopsies from macroscopically abnormal lesions in the TI, 44 (8.0%) had significant diseases. The frequency of significant disease was high in patients with clinically suspected inflammatory bowel disease (IBD) (50.0%), anemia (31.6%), right lower quadrant (RLQ) pain (28.6%), and radiological abnormalities in the TI (27.5%). The frequency of Crohn’s disease (CD) was high in patients with clinically suspected IBD. A concurrent abnormality in the ileocecal valve (ICV) (14.3%) and the presence of an ulcer (14.2%), mass, or polyp (25.4%) correlated with a high incidence of significant disease, particularly CD. In cases of suspected IBD, anemia, RLQ pain, and radiologic abnormalities in the TI, there is a high possibility of significant disease. Ulcers, masses, polyps, and concurrent abnormalities in the ICV were also associated with significant disease.

## 1. Introduction

Despite the recent widespread use of enteroscopy, it is still difficult to diagnose small intestine disease because enteroscopy is technically difficult, time-consuming, and painful to patients. Capsule endoscopy is a safe and effective method and may help diagnose small intestinal disease by obtaining an image of the lesion; however, histological examination is impossible [1]. We can indirectly obtain information on diseases affecting the small intestine by observing the terminal ileum (TI) during colonoscopy [2]. Terminal ileum intubation is commonly performed during colonoscopy with little additional time and no significant difficulty [3,4]. Recently, the detection rate of TI lesions has increased, with a success rate of 96–97% [5,6].

During ileoscopy, in many cases, there are no specific findings in the TI. Even if a lesion is found, it is more likely to be nonspecific rather than actually provide a clue for the diagnosis of a specific disease [6,7]. Thus, the diagnostic value of TI intubation during colonoscopy is not well established, and some controversy exists concerning routine TI intubation [6,8]. Previous studies demonstrated that biopsies performed on endoscopically normal TI are of low diagnostic value and, therefore, not recommended [9,10]. However, several studies have highlighted the necessity of biopsy when macroscopic abnormalities are found in the TI. According to Velidedeoglu et al., even if gross abnormal lesions are found in the TI during endoscopy, a biopsy is not required unless the patient is suspected of inflammatory bowel disease (IBD) [7]. Jain et al. found that biopsies of gross ulcers found in the TI helped diagnose specific diseases; however, biopsies of nonspecific lesions, including nodularities, should be avoided [11].

The ultimate purpose of ileal intubation is to identify lesions in the small intestine and diagnose the disease. Diseases suspected or diagnosed by TI intubation include Crohn’s disease (CD), drug-induced ileitis, Behçet’s disease, intestinal tuberculosis (TB), and tumors [2,12,13,14,15,16]. In addition, a previous study showed that identifying an abnormal ileal lesion during colonoscopy reduces the need for additional small bowel imaging in patients with clinically suspected CD [17]. A recent study reported that early diagnosis and proper treatment could result in better clinical outcomes for patients with CD [18], which highlights the role of TI evaluation during colonoscopy.

In the case of CD and intestinal TB, colonoscopy is the primarily recommended test with biopsy for abnormal lesions through observation of the entire large intestine including the TI. Both CD and TB enteritis form epithelial cell granuloma and cause intestinal inflammation at similar locations. However, as the treatment is completely different, it is important to differentiate both diseases [19]. In 2021, the number of new TB cases worldwide was 10.6 million (134 per 100,000 persons), an increase of 4.5% from the previous year (total 10.1 million, 130 per 100,000 persons). The number of deaths was 1.6 million, which was an increase of 6.7% from the previous year (1.5 million). As of 2021, South Korea still ranks first in the incidence of new TB cases (35.7 per 100,000 persons) and third in mortality (3.8 per 100,000 persons) among OECD member countries [20]. South Korea has been at the top of the list for more than 20 years since joining the OECD in 1996. Intestinal TB remains an important disease which needs to be differentiated from other intestinal diseases such as CD in South Korea. A previous study reported that findings which distinguish intestinal TB from CD include ileocecal valve opening and ileocecal lesions. In one study, ileocecal lesions appeared in 100% of intestinal TB and in 91% of CD [21]. However, another study showed that involvement of the ileocecal area was more frequent in CD in CT imaging (70% vs. 43%), suggesting that it is not easy to distinguish what kind of disease a patient actually has with ileoscopy alone [22].

Thus, the current study aimed to identify patients who require TI intubation and biopsy during colonoscopy. Additionally, we identified which diseases were actually diagnosed when ICV lesion were found with TI abnormality in a Korean study population with a high TB rate. We investigated the endoscopic indications and macroscopic abnormalities associated with the diagnosis of significant disease.

## 2. Patients and Methods

### 2.1. Study Population

This retrospective study included patients who underwent colonoscopies between February 2000 and June 2019 at the referral center of Konyang University Hospital. Data from TI biopsies during colonoscopies were retrieved from our colonoscopy registry. When a patient underwent multiple tests and biopsies during the study period, only the first abnormal endoscopic findings and biopsy results were included. In this process, 234 patients were excluded from the study. Exclusion criteria included patients whose endoscopic indications were not available (*n* = 327), normal ileal findings (*n* = 41), and colonoscopies without results records (*n* = 12). Pathologic results of nodular lymphoid hyperplasia (*n* = 69) were excluded from the results analysis. Finally, a total of 551 patients who underwent biopsy of abnormal lesions in the TI during colonoscopy (Olympus Optical Co., Ltd., Tokyo, Japan, CF-Q260AI, and CF-HQ290I) were included. Medical records were reviewed to obtain basic patient information, endoscopic indications, endoscopic findings, and biopsy results.

### 2.2. Clinical Indications of Colonoscopy

Abdominal pain in the right lower quadrant (RLQ) area was classified as RLQ pain. Abdominal pain in areas other than the RLQ or other cases that were not clearly described in the medical records were classified as other abdominal pain. Any abnormality in the TI identified in radiography, CT, or MRI was regarded as a radiologic abnormality. All changes, other than diarrhea or constipation, were classified as changes in bowel habits. 

### 2.3. Endoscopic Findings

Endoscopic findings were obtained from medical records. The presence of ulcers (larger and distinct from aphthous), aphthous ulcers or erosions, erythema, and edema were described. An ulcer was defined as the most severe finding, and erythema or edema was defined as the mildest finding. Only the most severe findings were analyzed. In addition, the presence of masses, polyps, or abnormal findings in the ileocecal valve were examined. In this study, there were no cases with the simultaneous presence of different lesions (e.g., ulcers and polyps). 

### 2.4. Biopsy Analysis

The biopsy results were compared with the indication for endoscopy and gross endoscopic findings. Significant disease in the biopsy was defined for those patients in which a specific disease was suspected or confirmed in the biopsy result and additional examination other than endoscopy or treatment for the disease was needed. These significant diseases included inflammatory bowel disease (CD, ulcerative colitis), intestinal TB, neoplastic diseases, infectious diseases, and Behçet’s disease. Biopsies with acute or chronic inflammation, erosion, ulceration, lymphoid hyperplasia, and nonspecific ileitis were classified as nonspecific diseases.

### 2.5. Statistical Analysis

Statistical analyses were performed using IBM SPSS version 25. Logistic regression analyses were performed to determine the associations between different variables and significant diseases. The results of the multiple logistic regression were provided by all factors as independent variables and are presented as odds ratios (OR) with 95% confidence intervals (CI). A *p*-value < 0.05 was considered statistically significant. An odds ratio was considered statistically significant when the associated CI did not include 1.0.

## 3. Results

In total, 551 patients (363 males (65.9%) and 188 females (34.1%)) were included in this study. The median age at the time of biopsy was 49.0 years (standard deviation [SD] = 15.3, range 10–91). 

### 3.1. Significant Disease Diagnosis Rate by Endoscopic Indication

A total of 240 colonoscopies (43.6%) were performed during medical checkups. The second most common indication (144 cases; 26.1%) was other abdominal pain (abdominal pain other than RLQ). The other indications were diarrhea (53 cases; 9.6%), bloody stools (43 cases; 7.8%), radiologic abnormalities (40 cases; 7.3%), post-polypectomy follow-up (27 cases; 4.9%), change in bowel habits (19 cases; 3.4%), anemia (19 cases; 3.4%), RLQ pain (14 cases; 2.5%), suspected IBD (12 cases; 2.2%), and constipation (3 cases; 0.5%). Overall, 44 (8.0%) of the 551 colonoscopies had significant disease (Table 1).

A total of 40 cases had radiologic TI abnormalities, of which 11 (27.5%) were specific diseases (5 malignant lymphoma; 3 CD; and 2 intestinal TB). Among the 19 colonoscopies performed to identify the cause of anemia, 6 (31.6%) found specific diseases (2 CD; 1 adenocarcinoma; 1 tubular adenoma). Among the 14 patients undergone the procedure due to RLQ pain, 4 (28.6%) had significant diseases (2 CD; 2 diffuse large B-cell lymphoma). A total of 12 colonoscopies were performed in patients with clinically suspected IBD. Among them, six cases (50.0%) showed specific diseases (six CD; one intestinal TB). Lastly, only 9 (3.8%) of the 240 colonoscopies performed for medical checkups were diagnosed with a significant disease.

### 3.2. Significant Disease Diagnosis Rate by the Macroscopic Finding of the Terminal Ileum

The most common gross abnormalities in the TI were aphthous ulcers and erosions, observed in 245 cases (44.5%). The second most common endoscopic finding was ulcers (155 cases, 28.1%). In addition, 88 cases had erythema or edema (16.0%), and 63 had masses or polyps (11.4%). A total of 70 cases (12.7%) were accompanied by abnormalities in the ileocecal valve (ICV) (24 ulcers, 23 aphthous ulcers or erosions, 19 erythema or edema, and 4 masses or polyps). None of the patients had more than two different endoscopic abnormalities (Table 2).

Biopsy results were analyzed by comparison with gross endoscopic findings in the TI. Among the 155 cases of TI ulcers, 22 (14.2%) were specific diseases and this association was statistically significant. Among the 22 cases, CD was the most common (16 cases), and the remainder comprised 5 cases of intestinal TB and 2 cases of malignant lymphoma (1 DLBCL and 1 mantle cell lymphoma) (Table 3). Of the 70 cases with concurrent abnormalities in the ICV, 10 (14.3%) were specific diseases (six cases of CD, two cases of intestinal TB, one case of DLBCL, and one case of Behçet’s disease). 

There were 63 cases of masses or polyps, of which 16 (25.4%) had specific diseases and this association was statistically significant. There were 10 cases of adenoma (tubular, 8; tubulovillous, 2), 4 cases of DLBCL, and 1 case of adenocarcinoma. Only 1 patient was diagnosed with CD. The incidence of significant disease was significantly lower (1.6%) when aphthous ulcers or erosions were observed.

## 4. Discussion

Terminal ileal intubation is a relatively safe and easily performed procedure during colonoscopy, does not require additional time, and can be used as an indicator for complete colonoscopy. It may also be useful when the assessment of IBD or a specific infection is needed or when radiologic findings reveal abnormalities in the TI [10]. However, the usefulness of routine TI intubation in all colonoscopies remains controversial, and the diagnostic value of biopsy for gross abnormal lesions in the TI is not well established. Previously, the clinical yield of routine ileal inspections during colonoscopy was less than 5%, ranging from 1–4.6% [8,23]. Further, a recent study showed that most TI ulcers observed incidentally during colonoscopy for asymptomatic subjects had no significant results on biopsy and spontaneously improved on follow-up colonoscopy [24]. 

However, based on the results of this study, the rate of significant disease was 8.0% in the biopsy results of the enrolled patients. With respect to endoscopic indications, in patients with radiologic abnormalities in the TI, anemia, RLQ pain, or clinically suspected IBD, the incidence of specific diseases was significantly high. CD was diagnosed most frequently in patients with anemia, RLQ pain, and suspected IBD. Neoplastic diseases, such as malignant lymphoma, were most frequent in patients with radiological abnormalities in the TI.

According to previous studies, when colonoscopy was performed for RLQ pain, specific histopathological results were present and this was statistically significant when compared with endoscopy for screening [6]. Borsch et al. reported that clinically valuable information can be obtained from TI intubation in patients with clinically or previously diagnosed IBD [25]. McHugh et al. recommended TI intubation and ileal biopsy for patients with TI abnormalities in imaging studies or clinically suspected IBD [9]. Another study by Koksal et al. reported that histopathological evaluation via ileoscopy of normal TI in patients with anemia and abdominal pain has diagnostic value [26]. In terms of endoscopic indications, our results are in close agreement with those of previous studies, which may lead to better outcomes in patients with CD through earlier diagnosis. Nodular lymphoid hyperplasia was first considered as one of the result variables. However, previous studies showed that lymphoid hyperplasia in the terminal ileum is usually found in children and is commonly associated with allergic or infectious disease, with a mostly benign course [27]. Previous papers demonstrated the importance of lymphoid hyperplasia in the intestine; however, its association was mostly with an immunological mucosal response such as allergy or infection [28,29]. In fact, our study results had 69 patients who showed only lymphoid hyperplasia in the terminal ileum; however, none of them were diagnosed with significant disease. Thus, we finally decided to exclude lymphoid hyperplasia from the result analysis.

Recently, a study showed TI biopsy results among patients who had macroscopic abnormalities during colonoscopy that were all CD [30]. In contrast, the current study revealed the rate and type of disease diagnosed in detail depending on the type of gross abnormality (Table 3). The incidence of diseases was high when the lesions of TI were ulcers. Previously, Mayank et al. also reported that biopsies may be helpful in the diagnosis of disease when the TI abnormality is an ulcer [11]. However, what is different about our study is that we classified ulcers into two types. Actually, our results showed that aphthous ulcers or erosions showed lower rates of significant disease. A possible explanation is that aphthous ulcers are usually nonspecific, benign, and can be found in Behçet’s disease, intestinal TB, amebic colitis, drug-induced colitis, and even in normal people [31]. Further, a prior study documented that none of the aphthous ulcers in TI investigated as part of their work had sole histologic abnormalities [9]. Another finding is that concurrent ICV lesions were also statistically significant and CD was the highest among the diagnosed diseases. Previous reports have shown that lesions in the ICV are typically seen in intestinal TB and relatively rare in CD [20,32]. However, based on our results, concerning concurrent ICV lesions, it is more likely to consider CD. Therefore, we suggest that biopsy of TI lesions should be performed in case of concurrent abnormalities in the ICV for the high possibility of CD as well as the distinction of intestinal TB.

In this study, we demonstrated the diagnostic yield of biopsies performed on gross abnormalities of the TI. Except for several specific endoscopic indications or gross findings revealed in this study, most abnormal lesions of the TI were of little clinical significance. However, a recent study reported that 34.3% of patients with isolated TI lesions were diagnosed as having CD [33]. Despite the possibility of bias as the proportion of patients who underwent capsule endoscopy was not described in detail, a large number of patients have been diagnosed with significant disease by capsule endoscopy. Due to the cross-sectional and retrospective characteristics of this study, there is a possibility that the ratio of significant disease could be underestimated as analyses of additional tests were not performed. It is expected that further application of diagnostic tools that can evaluate the entire small intestine, such as capsule endoscopy and CT/MR enterography and enteroscopy, will help improve the diagnosis rate for small intestine diseases. 

This study had several limitations. First, the endoscopic findings were not classified on a more objective basis. However, all examiners specialized in gastroenterology, and there was no significant difference in the judgment of lesions during colonoscopy. Second, because of its retrospective design, we could not survey the role of medications taken at the time of endoscopy, such as aspirin or nonsteroidal anti-inflammatory drugs that could affect lesions. Likewise, with respect to the rate of performing TI biopsies, we do not have information on the overall number of colonoscopy tests and detailed data were not available. Furthermore, we could not include patients with abnormal TI findings without biopsy. Third, despite the necessity of pathologic confirmation for diagnosis of intestinal disease, in clinical situations, final diagnoses are not made solely based on pathologic results. For example, in the case of intestinal TB, final diagnoses are made based on comprehensive exams including colonoscopy, biopsy, and acid-fast and radiologic tests. Still, in some cases, there might be a difficulty in distinction from Crohn’s disease. In this case, we made diagnoses by empirical use of anti-tuberculosis drugs. Last, although our sample size is large, due to the low incidence of patients with significant disease, further statistical analysis for subgroups is not available.

Nevertheless, this study has several strengths. First, as a long-term, large-scale study in an academic institute, our study provides valuable information on the performance of colonoscopy in patients with IBD. Based on a significant number of samples from South Korea, where a high incidence of IBD and tuberculosis still exists, CD is more likely to be diagnosed than intestinal TB in patients with TI abnormalities accompanied by concurrent ICV lesions. In addition, we demonstrated a higher rate of significant disease in the TI and evidence for the need of thorough inspection of ileal lesions during colonoscopy. Second, this study demonstrated in detail the types of diseases diagnosed according to each endoscopic indication and macroscopic finding. By providing updated diagnostic value of TI lesions, we believe our findings can help determine which patients should be further tested and establish endoscopic guidelines in clinical situations. 

In summary, for suspected IBD, anemia, RLQ pain, and radiologic abnormalities in the TI, there is a high possibility of significant disease. Ulcers, masses, polyps, and concurrent abnormalities in the ICV were also associated with significant disease. Further prospective, multicenter studies will be required in the future. 

## Figures and Tables

**Table 1:** Diagnostic yield of terminal ileal biopsy varied with the indication for colonoscopy.

Indication	*n*	Significant Diseases, *n* (%)	Univariate OR [95% CI]	Multiple OR [95% CI]
Screening	240	9 (3.8)	0.31 [0.14–0.65]	0.48 [0.14–1.63]
Abdominal pain (except RLQ)	144	14 (9.7)	1.353 [0.70–2.63]	0.84 [0.30–2.32]
Diarrhea	53	10 (18.9)	3.174 [1.47–6.86]	1.97 [0.65–5.95]
Hematochezia or melena	43	3 (7.0)	0.85 [0.25–2.88]	0.54 [0.13–2.33]
Radiologic abnormalities in TI	40	11 (27.5)	5.48 [2.52–11.94]	2.93 [1.01–8.45]
Post-polypectomy surveillance	27	0 (0.0)	0.92 [0.89–0.94]	-
Bowel habit change	19	1(5.3)	0.63 [0.08–4.85]	0.66 [0.07–5.88]
Anemia	19	6 (31.6)	5.99 [2.16–16.64]	4.50 [1.29–15.75]
RLQ pain	14	4 (28.6)	4.97 [1.49–16.56]	2.24 [0.53–9.42]
Clinically suspected IBD	12	6 (50.0)	13.18 [4.06–42.85]	5.28 [1.36–20.53]
Constipation	3	0 (0.0)	0.92 [0.90–0.94]	
All	551	44 (8.0)		

RLQ, right lower quadrant; OR, odds ratio; CI, confidence interval; IBD, inflammatory bowel disease; TI, terminal ileum.

**Table 2:** Diagnostic yield of biopsy varied with the endoscopic finding of the terminal ileum.

Findings	*n*	Significant Disease, *n* (%)	Univariate	Multiple
			OR [95% CI]	OR [95% CI]
Ulcer	155	22 (14.2)	2.81 [1.51–5.24]	0.43 [0.20–0.90]
Aphthous ulcer or erosion	245	4 (1.6)	0.11 [0.04–0.31]	0.04 [0.01–0.14]
Erythema or edema	88	2 (2.3)	0.23 [0.06–0.98]	0.05 [0.01–0.25]
Mass or polyp	63	16 (25.4)	5.59 [2.82–11.08]	-
Concurrent abnormality in ICV	70	10 (14.3)	2.191 [1.03–4.66]	2.72 [1.18–6.26]

OR, odds ratio; CI, confidence interval; ICV, ileocecal valve.

**Table 3:** Diagnosis according to endoscopic indications and macroscopic findings.

Variables	Crohn’s	Intestinal TB	Neoplastic	Behçet’s
**Indication**				
Screening	2	1	5	1
Abdominal pain	6	4	4	
Diarrhea	9		1	
Hematochezia or melena	1		2	
Radiologic abnormality	3	2	6	
Suspected IBD	5	1		
Bowel habit change	1			
Anemia	4		2	
RLQ Pain	2		2	
**Macroscopic finding**				
Ulcer	16	5	2	
Aphthous ulcer or erosion	3			1
Erythema or edema	1	1		
Mass or polyp	1		15	
Concurrent abnormality in ICV	6	2	1	1

RLQ, right lower quadrant; IBD, inflammatory bowel disease; ICV, ileocecal valve.

## Data Availability

Data can be made available from the corresponding author upon request.
